# Books and Magazines

**Published:** 1906-10

**Authors:** 


### Books and Magazines.

THE TRAGEDY OF BADEN. C. H. Wilkinson, M. D., Galveston. Texas. Cloth; gilt letters. Price, $1.25. Neale & Co., Publishers, New York.

This is quite a thrilling romance, based on actual occurrence. It is of a dapper young coffee drummer, who dropped into a

"reputable" saloon and took a glass of beer. The proprietor, a notoriously bad character, drugged the beer with chloral, robbed the young man and sent him to the hospital. He apparently died, and was buried within six hours. The young doctors of the hospital staff, in collusion with the sexton, dug him up for the purpose of dissection. Exposure to the air revived him, and he lived and brought the old robber to trial. The heroine—the pretty girl was an orphan at a convent. She was "adopted" by the old scoundrel and his wife, who secured her from the good sisters upon forged endorsements as to character, etc., when she was put to the most menial labors and finally was made to hand beer and play rag-time pieces on the piano for the rowdy frequenters of the saloon. She was rescued by a detective, a former lover of her mother. The story is full of action, and is well written. Interest never flags from start to finish. I congratulate my old friend, Prof. Wilkinson, upon this, his first, venture at fiction. The object of the book is to show the danger of too early burial.

---

APPLETON'S LATEST: A TEXT-BOOK OF HUMAN PHYSIOLOGY. By Dr. Robert Tigerstedt, Professor of Physiology in the University of Helsingsfors, Finland. Translated from the third German edition, and edited by John R. Murlin, A. M., Ph. D., Assistant Professor of Physiology in the University and Bellevue Hospital Medical College, New York City; with an introduction to the English edition by Professor Graham Lusk, Ph. D., F. R. S. (Edinburgh), New York and London. D. Appleton.

Aside from the general excellence of this book, which may be taken for granted in view of the author's reputation as a physiologist, it presents a number of noteworthy and commendable features which differ from those of any other text-book yet published. The first of these is the manner of approach to the real subject. After a chapter on General Method, in which the author gives some instances of how exact physiological knowledge is gained, he takes the pains to lay a very broad foundation to the special subject of human physiology. The second chapter is a very compact one on the Physiology of the Cell. This constitutes the biological foundation. The next is a chapter on the Chemical Constituents of the Body which supplies the chemical foundation, but does not carry the reader to a needless length into the vast field of physiological chemistry. The fourth chapter is devoted to Metabolism and Nutrition, and in it the author points out (for the first time, we believe, in any physiology) that all the energy transformations of the body rest on the principle of the conserva-

**The usual combination of impoverished blood, nervous exhaustion, lack of digestive vigor yields most quickly to**

### GRAY'S GLYCERINE TONIC COMP.

**It restores, nourishes, reconstructs; besides this, it is especially efficient in diseases of the Chest and Throat**

THE PURDUE FREDERICK CO.,
298 Broadway, New York.

tion of energy. He also describes the methods by which the very exact information of today, regarding the general value of the foodstuffs and the nutritive requirements of man under different circumstances, is gained. This constitutes the physical basis of human physiology.

Another feature worthy of special mention is the classification of the subject matter of the book. A clear and logical analysis underlies the order of presentation throughout. Following the introductory chapters just enumerated, the special subject of human physiology begins with a chapter on the blood.

Every chapter of the book is as complete as possible within the limits of a single volume, and many of them are very originally conceived. This is especially true of the chapter on Metabolism and Nutrition.

The chapter on the Circulation and the one on Metabolism and Nutrition fall within the author's own chosen fields of investigation. Needless to say they contain much that is new and of great importance.

In his treatment of the Physiology of the Nervous System, the author reaches the climax of the book, whether we consider it from the standpoint of the practitioner of medicine or of the general reader.

We should not neglect to mention the great number of excellent illustrations. There are 305 illustrations in this work, 63 of which are in colors. We believe it to be the best illustrated text-book upon this subject yet published.

Altogether, this translation of Tigerstedt's Text-Book of Physiology is, we believe, the most complete and most broadly conceived one for its size in any language. Its appearance in English will, no doubt, meet with the welcome it deserves.

---

A Non-Surgical Treatise on Diseases of the Prostate Gland and Adnexa. By Geo. Whitfield Overall, A. B., M. D., Chicago. Rowe Publishing Co., 1906. Cloth; p. 225.

The author states in the preface that in less than a year the second double edition of this work is exhausted, (I don't know what a double edition is) and that the "flattering criticisms of the medical press" and the "almost universal commendations of the readers, evinces the fact that the little book has filled the void for which it was intended." That is the *raison d'etre,* and he has said it better than I could have done. But it seems to me that, incidentally, it was intended to advertise and sell certain instruments and devices of the author's invention (illustrated), the use of which, in treatment, could hardly be called "non-surgical." It is worth $1; price not stated.

